# Supplementary material for: The Association of Active Living Environments and Mental Health: A Canadian Epidemiological Analysis
Source: Int J Environ Res Public Health. 2020 Mar 15;17(6):1910. doi: 10.3390/ijerph17061910 (PMC7142646; doi:10.3390/ijerph17061910)
Supplement: Supplementary file 1 [file ijerph-17-01910-s001.pdf]

Table S1: Crude Models of Mental Health Outcomes and Obesity

Exposure	PHQ-9 10+		PHQ-9 5+		Mood Disorder		Anxiety Disorder		Obesity	
**ALE**	OR (95% CI)	p-value	OR (95% CI)	p-value	OR (95% CI)	p-value	OR (95% CI)	p-value	OR (95% CI)	p-value
Class 1	0.82 (0.64-1.05)	0.12	0.77 (0.67-0.89)	<0.01	0.94 (0.82, 1.07)	0.36	1.08 (0.95, 1.23)	0.24	1.86 (1.66-2.08)	<0.01
Class 2	0.80 (0.62-1.04)	0.10	0.85 (0.73-0.99)	0.03	1.00 (0.88, 1.51)	0.90	1.08 (0.95, 1.23)	0.25	1.61 (1.44-1.82)	<0.01
Class 3	0.97 (0.74-1.27)	0.81	0.87 (0.75-1.02)	0.10	1.02 (0.89, 1.17)	0.78	1.14 (0.99, 1.30)	0.06	1.42 (1.26-2.61)	<0.01

Table S2: Models of Mental Health Outcomes adjusted for potential confounders

Exposure	PHQ-9 10+		PHQ-9 5+		Mood Disorder		Anxiety Disorder	
	OR (95% CI)	p-value	OR (95% CI)	p-value	OR (95% CI)	p-value	OR (95% CI)	p-value
Active Living Environment								
Class 1	0.98 (0.71-1.35)	0.90	0.91 (0.76-1.10)	0.33	0.88 (0.74, 1.05)	0.16	0.95 (0.80, 1.13)	0.56
Class 2	0.92 (0.67-1.25)	0.59	0.97 (0.81-1.16)	0.73	0.93 (0.79, 1.09)	0.36	0.98 (0.84, 1.16)	0.84
Class 3	1.11 (0.80-1.54)	0.53	0.96 (0.79-1.16)	0.67	0.95 (0.80, 1.12)	0.51	1.01 (0.85, 1.19)	0.94
Material Deprivation								
Quintile 2	0.93 (0.72-1.19)	0.56	0.96 (0.83-1.11)	0.54	0.89 (0.78, 1.01)	0.07	1.07 (0.93, 1.22)	0.34
Quintile 3	0.85 (0.67-1.07)	0.15	0.87 (0.75-1.00)	0.05	1.04 (0.92, 1.19)	0.52	1.17 (1.02, 1.34)	0.02
Quintile 4	0.95 (0.74-1.22)	0.71	0.96 (0.82-1.11)	0.57	1.01 (0.89, 1.15)	0.82	1.30 (1.13, 1.49)	<0.01
Quintile 5	1.00 (0.78-1.28)	1.00	0.93 (0.80-1.09)	0.38	0.93 (0.82, 1.07)	0.32	1.39 (1.22, 1.59)	<0.01
Social Deprivation								
Quintile 2	1.37 (1.07-1.77)	0.01	1.10 (0.95-1.27)	0.20	1.15 (0.99, 1.34)	0.07	1.16 (0.98, 1.37)	0.08
Quintile 3	1.51 (1.17-1.93)	<0.01	1.07 (0.91-1.25)	0.40	1.14 (0.98, 1.33)	0.09	1.22 (1.04, 1.43)	0.02
Quintile 4	1.61 (1.29-2.01)	<0.01	1.28 (1.11-1.48)	<0.01	1.25 (1.08, 1.45)	<0.01	1.20 (1.03, 1.40)	0.02
Quintile 5	2.05 (1.61-2.59)	<0.01	1.46 (1.26-1.70)	<0.01	1.50 (1.29, 1.75)	<0.01	1.45 (1.24, 1.69)	<0.01
Has a Chronic Condition	2.92 (2.47-3.46)	<0.01	2.30 (2.08-2.53)	<0.01	2.92 (2.65, 3.21)	<0.01	3.17 (2.87, 3.51)	<0.01
Age (12+)	0.98 (0.97-0.98)	<0.01	0.98 (0.97-0.98)	<0.01	0.98 (0.98, 0.99)	<0.01	0.97 (0.97, 0.98)	<0.01
Sex	1.87 (1.61-2.17)	<0.01	1.39 (1.27-1.53)	<0.01	1.75 (1.61, 1.90)	<0.01	1.81 (1.67, 1.97)	<0.01
Marital Status								
Single	1.44 (1.19-1.74)	<0.01	1.29 (1.14-1.45)	<0.01	1.30 (1.17, 1.45)	<0.01	1.20 (1.08, 1.34)	<0.01
Divorced	1.74 (1.43-2.12)	<0.01	1.47 (1.30-1.67)	<0.01	1.93 (1.72, 2.16)	<0.01	1.56 (1.38, 1.76)	<0.01
Highest Level of Education								
No Secondary Degree	1.33 (1.08-1.64)	<0.01	1.22 (1.07-1.40)	<0.01	0.89 (0.80, 0.99)	0.04	1.01 (0.90, 1.14)	0.889
No Post-Secondary Education	1.33 (1.13-1.56)	<0.01	1.21 (1.09-1.35)	<0.01	1.00 (0.91, 1.10)	0.95	1.16 (1.05, 1.28)	<0.01
Employment (Last Week)								
Absent	1.66 (1.29-2.13)	<0.01	1.47 (1.22-1.77)	<0.01	1.73 (1.46, 2.05)	<0.01	1.37 (1.14, 1.63)	<0.01
No job	1.51 (1.30-1.76)	<0.01	1.24 (1.12-1.38)	<0.01	1.71 (1.56, 1.87)	<0.01	1.50 (1.36, 1.65)	<0.01
Income Quartile								
Quartile 1	1.44 (1.17-1.78)	<0.01	1.42 (1.26-1.61)	<0.01	1.45 (1.29, 1.64)	<0.01	1.32 (1.17, 1.48)	<0.01
Quartile 2	1.28 (1.04-1.56)	0.02	1.06 (0.94-1.19)	0.32	1.07 (0.95, 1.20)	0.27	1.02 (0.91, 1.14)	0.79
Quartile 3	1.16 (0.94-1.43)	0.18	0.99 (0.88-1.11)	0.88	1.04 (0.92, 1.17)	0.52	1.08 (0.96, 1.22)	0.188
Immigrant	0.75 (0.61-0.92)	<0.01	0.90 (0.80-1.02)	0.11	0.53 (0.47, 0.60)	<0.01	0.46 (0.40, 0.53)	<0.01
BMI as Obese	1.62 (1.40-1.88)	<0.01	1.42 (1.29-1.56)	<0.01	1.55 (1.42, 1.69)	<0.01	1.16 (1.07, 1.27)	<0.01

Table S3: Model of Obesity as an Outcome (Full)

Exposure	Obesity	
	OR (95% CI)	p-value
Active Living Environment		
Class 1	1.65 (1.43-2.03)	<0.01
Class 2	1.47 (1.20-1.81)	<0.01
Class 3	1.21 (0.97-1.50)	0.09
Material Deprivation		
Quintile 2	1.32 (1.14-1.53)	<0.01
Quintile 3	1.32 (1.15-1.52)	<0.01
Quintile 4	1.68 (1.45-1.94)	<0.01
Quintile 5	1.83 (1.57-2.13)	<0.01
Social Deprivation		
Quintile 2	1.20 (1.05-1.37)	<0.01
Quintile 3	1.26 (1.11-1.44)	<0.01
Quintile 4	1.25 (1.10-1.42)	<0.01
Quintile 5	1.23 (1.07-1.40)	<0.01
Has a Chronic Condition	1.82 (1.66-1.99)	<0.01
Age (12+)	1.01 (1.01-1.01)	<0.01
Sex	0.77 (0.71-0.83)	<0.01
Marital Status		
Single	0.70 (0.62-0.80)	<0.01
Divorced	0.88 (0.78-0.99)	0.04
Highest Level of Education		
No Secondary Degree	1.07 (0.94-1.21)	0.30
No Post-Secondary Education	1.15 (1.05-1.26)	<0.01
Employment (Last Week)		
Absent	1.14 (0.96-1.36)	0.12
No job	0.86 (0.78-0.95)	<0.01
Income Quartile		
Quartile 1	1.04 (0.91-1.18)	0.57
Quartile 2	0.99 (0.89-1.10)	0.86
Quartile 3	1.01 (0.91-1.13)	0.85
Immigrant	0.67 (0.59-0.75)	<0.01
PHQ-9 score (continuous)	1.04 (1.03-1.05)	<0.01
